# Potential for Collider Bias in Studies Examining the Association of Central Corneal Thickness With Glaucoma

**DOI:** 10.1167/iovs.63.12.3

**Published:** 2022-11-02

**Authors:** Anthony P. Khawaja, Nomdo M. Jansonius

**Affiliations:** 1 NIHR Biomedical Research Centre at Moorfields Eye Hospital NHS Foundation Trust & UCL Institute of Ophthalmology, London, United Kingdom; 2 Department of Ophthalmology, University of Groningen, University Medical Center Groningen, Groningen, The Netherlands

**Keywords:** glaucoma, risk factors, central corneal thickness, collider bias, simulations

## Abstract

**Purpose:**

Central corneal thickness (CCT) may be biologically related to glaucoma or observed as associated with glaucoma simply due to its effect on intraocular pressure (IOP) measurement. We aimed to determine if the previously reported CCT-glaucoma associations, in which the analyses were adjusted for IOP or participants were selected on IOP, could be explained by collider bias.

**Methods:**

We simulated datasets mimicking a longitudinal population-based study (Los Angeles Latino Eye Study) and a trial (Ocular Hypertension Treatment Study) such that: (i) CCT was not truly associated with glaucoma, (ii) CCT and true IOP both contribute to measured IOP, and (iii) true IOP contributes to glaucoma risk. We then tested whether an association between CCT and glaucoma could be spuriously induced simply by adjusting for or selecting on measured IOP.

**Results:**

A thinner CCT was significantly associated with higher glaucoma incidence in the simulated longitudinal population-based study when adjusted for measured IOP, but not crudely (unadjusted). A thinner CCT was crudely associated with glaucoma incidence in the simulated trial in which the participants were selected for high measured IOP. Effect sizes in the simulations were similar to those observed in the original studies.

**Conclusions:**

Our findings question whether CCT is biologically associated with glaucoma and suggest that current evidence may be due to collider bias. This indicates that CCT alone cannot be used as a factor to identify people at high risk of glaucoma in the general population. Using CCT in combination with IOP may be superior to using IOP alone.

Glaucoma remains the leading cause of irreparable blindness globally.^1^ Given its irreversible nature, early diagnosis and avoidance of late presentation are key to preventing blindness. Whereas general population screening for glaucoma is not recommended as current tests perform inadequately with the relatively low disease prevalence, targeted screening of high-risk individuals may be cost-effective.^2^ Therefore, understanding the factors that increase the risk of glaucoma within populations is important to inform future screening and prevention strategies. Established risk factors for glaucoma include older age,^3^ higher intraocular pressure (IOP),^4^ non-White ethnicity,^3^ and a family history of glaucoma,^5^ and these factors may be used to identify enriched subsets of the population to target screening towards. Other potential risk factors for glaucoma remain less well-established, including a thin central corneal thickness (CCT).

CCT may be related to glaucoma in at least two different ways. First, CCT spuriously influences the measurement of IOP.^6^ Specifically, a particularly thick or thin CCT results in a departure from the assumptions of the Imbert Fick principle, such that eyes with a thinner CCT spuriously have lower IOP readings on average, and vice versa.^7^ Second, it has been postulated that CCT may be a biomarker of the biomechanical properties of the lamina cribrosa and surrounding tissues,^8^ and may therefore reflect susceptibility of the optic nerve head to damage from stress and strain induced by high IOP. Until now, it remains uncertain whether the spurious influence of CCT on IOP may fully explain the previously reported associations between CCT and glaucoma-related parameters in numerous studies, or whether the relationship is explained by a true biological link.^9^

A number of landmark glaucoma studies have reported association with CCT (see the Table). Jiang and colleagues reported a thinner baseline CCT to be associated with an increased risk of incident open-angle glaucoma (OAG) in the Los Angeles Latino Eye Study (LALES).^10^ However, CCT was not crudely associated with incident OAG (odds ratio [OR] per 40 µm thinner = 1.16, 95% confidence interval [CI] = 0.90–1.50, *P* = 0.25) and it was only in a multivariable model that included IOP that CCT became significantly associated. Similarly, a thinner CCT was associated with a higher risk of POAG progression in the Early Manifest Glaucoma Trial (EMGT), but – again – only in a multivariable analysis adjusted for IOP (hazard ratio [HR] per 40 µm thinner = 1.25, 95% CI = 1.01–1.55, *P* = 0.042); CCT was not associated with progression in an unadjusted model (HR for CCT <548.4 µm vs. ≥548.4 µm 1.23, 95% CI = 0.90–1.68; *P* = 0.19).^11^ The fact that, in both of these studies, CCT was not crudely associated and only became significant in multivariable analyses suggests that it is the adjustment for IOP that created the apparent association. Adjusting for a variable in a model is equivalent to holding that variable constant while estimating the other parameters. Variation in CCT while holding measured IOP constant may in turn reflect variation in true IOP. For example, if two patients had the same measured IOP of 15 mm Hg, but one had a CCT of 420 µm and the other a CCT of 620 µm, the CCT may indicate a difference in true IOP between these patients (the patient with a CCT of 420 µm likely has a higher true IOP than the patient with a CCT of 620 µm).

**Table.** Studies Reporting the Association Between Central Corneal Thickness (CCT) and Conversion From Ocular Hypertension (OHT) to Primary Open-Angle Glaucoma (POAG), Incidence of Open-Angle Glaucoma (OAG), or Progression of POAG

Study Name	Outcome Variable	Selected on IOP?	Significant Unadjusted Association With CCT?	Significant Iop-Adjusted Association With CCT?
Ocular Hypertension Treatment Study^12^	Conversion from OHT to POAG	Yes	Yes (HR per 40 µm thinner = 1.88; CI = 1.55-2.29; *P* < 0.05)	Yes (HR per 40 µm thinner = 1.71; CI = 1.40-2.09; *P* < 0.05)
Los Angeles Latino Eye Study^10^	Incident OAG	No	No (OR per 40 µm thinner = 1.16; CI = 0.90-1.50; *P* = 0.25)	Yes (OR per 40 µm thinner = 1.30; CI = 1.00-1.70; *P* = 0.050)
Early Manifest Glaucoma Treatment Study^11^	POAG progression	No	No (HR for CCT <548.4 µm vs. ≥548.4 µm 1.23; CI = 0.90–1.68; *P* = 0.19)	Yes (HR per 40 µm thinner = 1.25; CI = 1.01-1.55; *P* = 0.042)

IOP, intraocular pressure; HR, hazard ratio; OR, odds ratio per 40 µm decrease in CCT; 95% CI, 95% confidence interval.

A thinner CCT was associated with a higher risk of conversion from ocular hypertension (OHT) to POAG in the Ocular Hypertension Treatment Study (OHTS).^12^ Although this may suggest that CCT is a risk factor for POAG, it should be noted that the OHTS cohort was selected on measured IOP (24–32 mm Hg in one eye and 21–32 mm Hg in the fellow eye). Therefore, a thick CCT in this cohort may represent participants with a true IOP lower than the selection criteria, but with a spuriously higher measured IOP meeting the selection criteria. In other words, a thick CCT may be a marker of people with lower true IOP than the rest of the cohort and it is this artifact that has driven the association of CCT with conversion risk.

Based on our interpretation of the abovementioned studies, we hypothesize that CCT may not be truly associated with POAG, but that adjustment for or selection on measured IOP may induce an apparent association with CCT due to CCT then reflecting variation in true IOP. In the causal epidemiology literature, this type of spuriously induced association is known as a collider bias.^13^ Specifically, a collider bias occurs when adjusting for or selecting on a factor that both the exposure and the outcome cause (Fig. 1); in this situation, a spurious relationship between exposure and outcome can be created even if they are unrelated. This is in contrast to a confounding factor (a factor that causes both exposure and outcome); unadjusted associations will be biased and adjusting for the confounder will make the estimate less biased (see Fig. 1). Confounding factors are often well-recognized; a common confounding factor in many associations is age. Collider bias, on the contrary, is less often considered. An example of collider bias is the spurious, protective association between being a healthcare worker and the severity of a coronavirus disease 2019 (COVID-19) infection.^14^ When resources are limited, selection on testing occurs (with testing limited to healthcare workers and hospitalized patients from the general population); being tested therefore indicates either being a healthcare worker with any possible COVID-19 severity or a non-healthcare worker with severe disease, resulting in the observed spurious protective effect (applying this example to Fig. 1, “exposure” is being a healthcare worker, “outcome” is severe disease, and the “collider” is COVID-19 testing). Figure 2 presents how collider bias can occur when examining the association between CCT and glaucoma. Both CCT (the exposure) and true IOP (the outcome) contribute to (or cause) measured IOP (the collider); this means a spurious association can be created between CCT and true IOP if the analysis is adjusted for (e.g. LALES and EMGT) or selected on (e.g. OHTS) measured IOP. This, in turn, will create a spurious relationship between CCT and glaucoma that is mediated by true IOP (given the strong relationship between true IOP and glaucoma).

**Figure 1.**
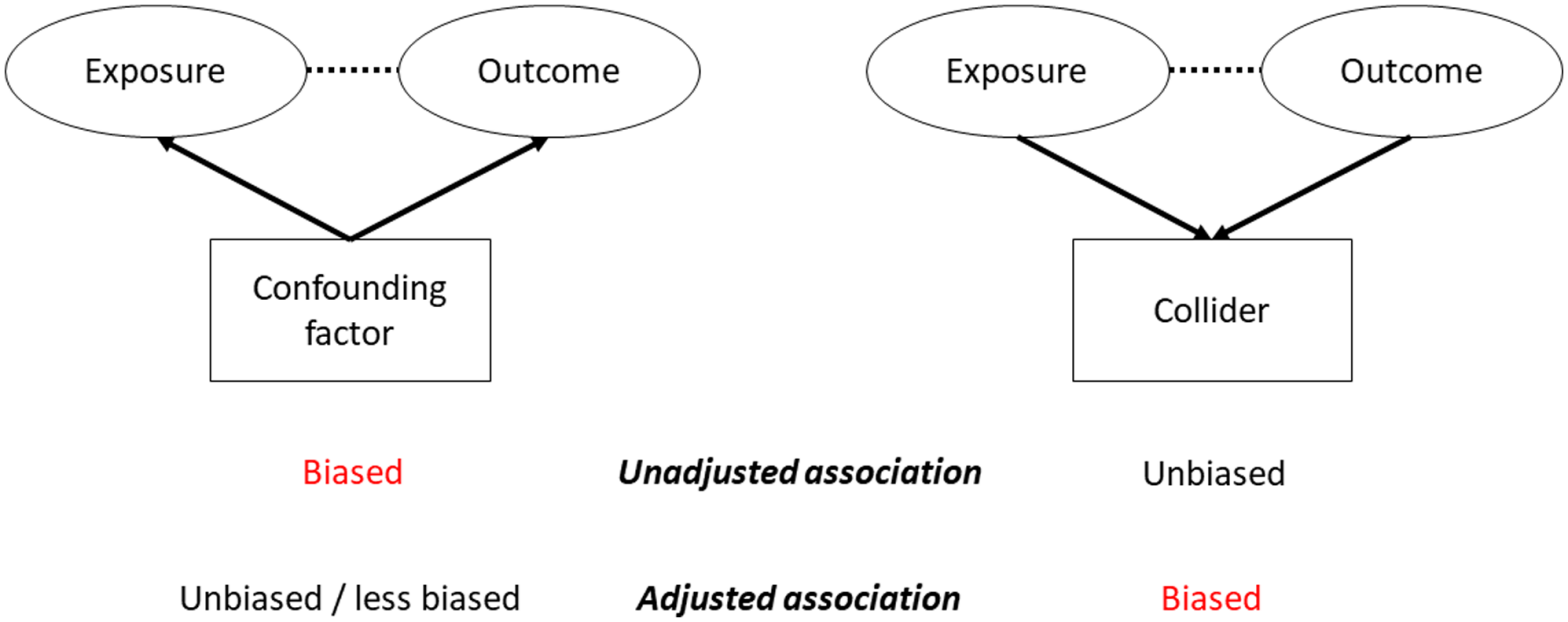
Directed acyclic graphs demonstrating confounding (*left*) and collider (*right*) bias. *Solid arrows* indicate a true causal relationship in the direction of the *arrow*. In this example, the exposure is not truly associated with the outcome. A biased, spurious association may occur between the exposure and outcome (*dashed line*) if there are any confounders **not** adjusted for, or if there are any colliders that are adjusted for, or selected on.

**Figure 2.**
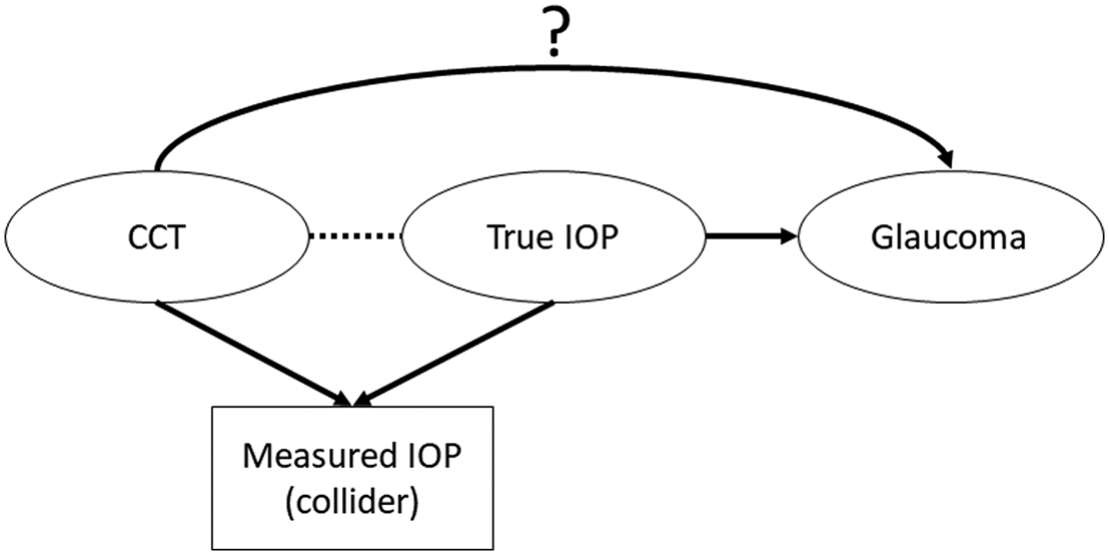
Directed acyclic graph demonstrating the potential role of collider bias when examining the association between central corneal thickness (CCT) and glaucoma (the hypothesized causal association is represented by the *arrow with a question mark*). Both CCT and true IOP contribute to (or *cause*) measured IOP (*solid arrows*), which means that measured IOP is a collider. A spurious relationship (*dashed line*) between CCT and true IOP (and then glaucoma, in turn) will be created if the analysis is selected on or adjusted for measured IOP. CCT is the exposure, true IOP is the outcome and measured IOP is the collider. The spurious relationship between CCT and glaucoma, induced by the collider bias, is mediated via true IOP.

Clearly, we are unable to prove whether our collider bias hypothesis is the cause for the associations with CCT in LALES, EMGT, or OHTS, as we will never be certain whether there is in fact a true association between CCT and glaucoma. Therefore, to test our hypothesis, we generated simulation datasets where we could control the relationship between the variables. We simulated datasets such that: (i) CCT was not truly associated with glaucoma, (ii) CCT and true IOP both contribute to measured IOP, and (iii) true IOP contributes to glaucoma risk. We then tested whether an association between CCT and glaucoma could be spuriously induced simply by adjusting for or selecting on measured IOP. To allow for a quantitative evaluation of the collider-bias effects, we made the characteristics of the variables in our simulated datasets as equal as possible to that of LALES (for evaluating the effect of adjusting for measured IOP) and OHTS (for evaluating the effect of selection on measured IOP). We then discuss our findings in relation to LALES, EMGT, and OHTS to infer whether their reported CCT associations may be due solely to collider bias.

## Methods

Simulated datasets were generated using the *rand* (uniformly distributed random numbers) and *randn* (normally distributed random numbers) function in Octave (version 5.2.0 for Ubuntu Linux 20.04; www.octave.org). Each dataset had the following variables included: age, CCT, measured IOP (IOPm), and true IOP (IOPt). Two different series of datasets were generated. The first series consisted of 20 simulated population-based datasets randomly generated from distributions mimicking LALES descriptive statistics.^10^ These datasets had a uniformly distributed age between 40 and 80 years, a normally distributed CCT with mean (standard deviation [SD]) of 551 (35) µm, and a sample size of 3939 participants per simulated dataset. The second series consisted of 10 simulated datasets randomly generated from distributions mimicking OHTS descriptive statistics.^15^ Here, we started with 10 population-based datasets with a sample size of 300,000 participants each. Again, we used a uniformly distributed age between 40 and 80 years; for CCT, we used a somewhat lower mean (540 µm) and greater SD (40 µm) to account for the mixed origin (25% African descent) of the OHTS participants. Those with an IOPm of (rounded) 24 mm Hg or higher were selected, yielding datasets of approximately 1600 “OHTS participants” each.

True IOP is not known. To circumvent this, we assumed the overall IOPt distribution to be equal to the IOPm distribution in the general population, and approached this distribution with a normal distribution with a mean (SD) of 15 (3) mm Hg.^16^ Starting with IOPt, we derived IOPm for each participant using a previously reported linear combination of IOPt and CCT^17^:

(1) $$\begin{array}{c} \mathrm{IOPm}=\mathrm{IOPt}-23.28+0.0423*\mathrm{CCT}\; \end{array}$$

Subsequently, we calculated, for each participant, an IOP-related glaucoma risk by assuming an OR of 1.18 per mm Hg of IOPt^10^^,^^15^^,^^18^ and an age-related glaucoma risk by assuming an OR of 1.08 per year of age.^10^^,^^18^^–^^20^ For participant *i*:

(2) $$\begin{array}{c} \mathrm{IOP}-\mathrm{related}\;\mathrm{risk}\left(i\right)=1.18^{(\mathrm{IOPt}\left(i\right)-<\mathrm{IOPt}>)}\; \end{array}$$

and

(3) $$\begin{array}{c} \mathrm{age}-\mathrm{related}\;\mathrm{risk}\left(i\right)=1.08^{(\mathrm{age}\left(i\right)-<\mathrm{age}>)}\; \end{array}$$

where <IOPt> and <age> are the mean IOPt and age, respectively, of a reference population.

For each participant, we calculated an overall glaucoma risk by multiplying a baseline risk (same value for all participants) by the individual IOP-related and age-related glaucoma risk. The baseline risk essentially equals the prevalence of glaucoma in a population with mean IOP and age equaling <IOPt> and <age>, respectively. In the simulations, we used <IOPt> = 15 mm Hg and <age> = 60 years. For baseline risk, we took the glaucoma prevalence at baseline for LALES (167/3939 = 0.042)^10^; for OHTS this number is unknown – we assumed the baseline risk to be 0.03.^21^ The overall glaucoma risk was subsequently compared to a random number allotted to the participant, taken from a uniform distribution between 0 and 1. If the random number was below the overall glaucoma risk, the participant was considered to have glaucoma at baseline and was excluded from the analysis (mimicking the baseline exclusion criteria for LALES and OHTS). Subsequently, we added another 4 or 5 years of age-related risk to the overall glaucoma risk of the remaining participants (mimicking the follow-up duration of 4 and 5 years in LALES^10^ and OHTS,^15^ respectively). If now the random number was below the updated overall glaucoma risk, the participant was considered to have incident glaucoma. Finally, by comparing those with and without incident glaucoma, we calculated the crude association with CCT and the association after adjusting for IOPm, using logistic regression (R version 3.6.3 for Ubuntu Linux 20.04; www.R-project.org). Results are presented as OR with 95% CI. In order to align with the literature, the ORs for CCT were presented as the effect per 40 µm decrease in CCT, that is, an OR above 1 would indicate a harmful effect of a thinner cornea.

## Results

**Figure 3.**
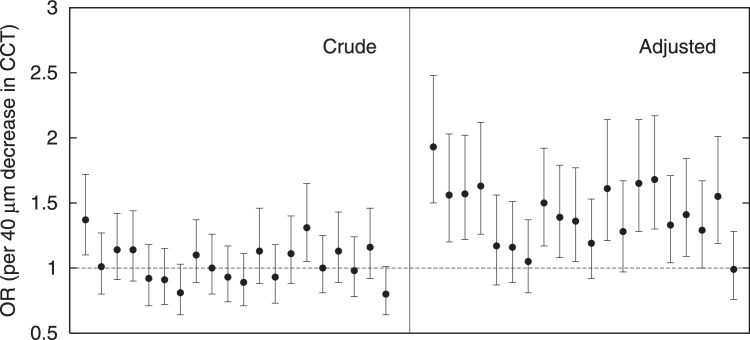
Central corneal thickness (CCT) as a risk factor for incident glaucoma in the general population. Results from the 20 simulations of LALES, both as the crude odds ratio (OR) for CCT (that is, without adjustment; *left panel*) and as the OR for CCT after adjusting for measured IOP (IOPm; *right panel*).

Figure 3 presents the results of the 20 simulations of the longitudinal population-based scenario (LALES), both as the crude OR of CCT (that is, without adjustment; left panel) and the OR of CCT after adjusting for IOPm (right panel). For the crude ORs, all but 2 of the 95% CIs included OR = 1. This aligns with what we would expect due to chance (95% CI represents a significant level of 5%; with 20 repeats, on average 1 [range = 0 to 5.6; Poisson distribution] significant result is to be expected by chance). For the adjusted ORs, all simulations showed a point-estimate OR greater than 1 (indicating a [spurious] harmful effect of a thin cornea) and the ORs of 14 of 20 simulations were significantly greater than 1 (*P* < 0.05). The mean (standard deviation [SD]) adjusted OR of the 20 simulations was 1.42 (0.24) per 40 µm decrease in CCT. The mean IOPm of the glaucoma cases was 16.5 mm Hg with a corresponding mean SD of 3.4 mm Hg; the mean CCT of the glaucoma cases was 550 µm with a corresponding mean SD of 34 µm. The mean 4-year incidence of glaucoma in the 20 simulations was 0.026 (on average, 96 new cases in 3681 participants).

**Figure 4.**
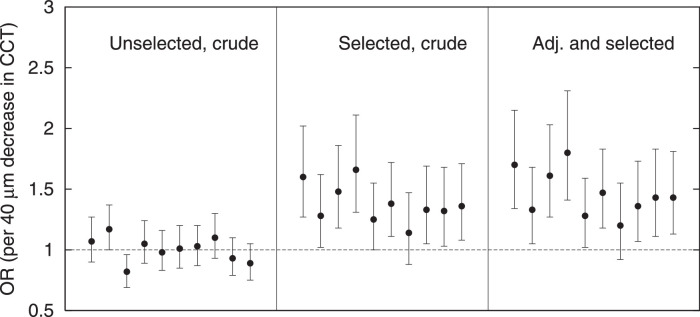
Central corneal thickness (CCT) as a risk factor for conversion to glaucoma in patients with ocular hypertension. Results from the 10 simulations of OHTS (i.e. participants selected for measure IOP [IOPm] ≥ 24 mm Hg), both as the crude odds ratio (OR) for CCT (that is, without adjustment; *middle panel*) and the OR for CCT after additional adjusting for IOPm (*right panel*). As a reference, we added the crude OR for CCT for 10 subsets of 6000 participants each, now randomly selected from the underlying datasets of 300,000 participants instead of selected on IOPm (*left panel*; with 6000 participants per subset we aimed for roughly the same number of incident glaucoma cases as in the scenario with selection).

Figure 4 presents the results for the 10 simulations of the incident glaucoma (also termed “conversion to glaucoma”) scenario in participants with OHTS, that is, in participants selected on IOPm. The figure shows both the crude ORs for CCT (middle panel) and the ORs for CCT after adjusting for IOPm (right panel). In the unadjusted analyses, thinner CCT was significantly associated with a higher risk of glaucoma in 9 out of 10 simulations (*P* < 0.05); the mean (SD) crude OR of the 10 repeats was 1.38 (0.16) per 40 µm decrease in CCT. In the analyses adjusted for IOPm, a very similar pattern was found, but with slightly higher point estimates; the mean (SD) crude OR of the 10 simulations was 1.46 (0.19) per 40 µm decrease in CCT. This modest additional effect of adjusting for IOPm is presumably related to the small IOPm range left after the initial selection (IOPm minimum-maximum is 24–32 mm Hg, but P2.5–P97.5 is only 24–27 mm Hg). The mean IOPm of the participants included in this longitudinal analysis was 24.6 mm Hg with a corresponding mean SD of 1.0 mm Hg; the mean CCT of the participants was 599 µm with a corresponding mean SD of 35 µm. The mean 5-year incidence of glaucoma in the 10 simulations was 0.086. As a reference, we added the crude OR of CCT for 10 subsets of 6000 participants each, now randomly selected from the underlying datasets of 300,000 participants, instead of selected on IOPm (see Fig. 4, left panel). In these analyses, only one simulation (the third) showed a clearly significant (*P* < 0.05) association between CCT and glaucoma, as expected just by chance. However, it had the opposite direction of effect as (OR < 1 versus OR > 1), reinforcing the absence of a real effect. The second simulation was borderline significant (*P* = 0.050).

## Discussion

CCT was significantly associated with glaucoma incidence in a simulated longitudinal population-based study when adjusted for measured IOP, but not crudely. Additionally, CCT was crudely associated with glaucoma incidence in a simulated study in which the participants were selected on measured IOP. These findings indicate the presence of collider bias, and this collider bias might explain the observed “independent” harmful association of a thin cornea in population-based studies and randomized controlled glaucoma trials.

In LALES, the observed adjusted OR for CCT was 1.30 per 40 µm decrease in CCT (see the Table). This value aligns well with the results of the simulations (see Fig. 3, right panel). The crude OR for CCT was not significant in LALES, which also agrees with our simulations (see Fig. 3, left panel). In OHTS, the observed crude and adjusted OR were 1.88 and 1.71 per 40 µm decrease in CCT, respectively (see the Table). These values are in the range of the simulations as well (see Fig. 4, middle and right panels), although somewhat at the upper edge. Hence, the simulations of both LALES and OHTS do not only qualitatively but also quantitatively agree with the original observations, which further supports our hypothesis that collider bias may spuriously induce an association between CCT and glaucoma in two scenarios: adjusting for or selecting on measured IOP. Following parsimony, it is plausible that the observed significant CCT-glaucoma associations in LALES and OHTS were solely due to this collider bias. “Mutatis mutandis” the same may be the case in EMGT. The fact that the crude associations in both LALES and EMGT were not significant further weakens the concept of a biological association between CCT and glaucoma. It is not possible to exclude the possibility of small biological effects though.

Another important approach to assessing causality of associations is Mendelian randomization. If an exposure is causally associated with an outcome, then we would expect genetic factors robustly associated with the exposure to also associate with the outcome in turn. Given the random allocation of genotype at conception, this approach is considered analogous to a randomized controlled trial and therefore less susceptible to biases due to confounding and reverse causation. Choquet and colleagues have conducted the largest genomewide association study for CCT to date, identifying nearly 100 significant independent loci.^22^ Using this data in a Mendelian randomization experiment, the investigators did not find evidence for a causal relationship between CCT and POAG.^22^ This is in agreement with our suggestion that CCT has only been previously associated with POAG due to collider bias, rather than a true biological link.

Simulations are a simplification of reality. Especially in the simulation of OHTS, we ignored the existence of two arms (one treated and one untreated), the different inclusion criteria for both eyes (one eye had to have an IOP between 24 and 32 mm Hg and the other eye between 21 and 32 mm Hg), and the fact that the IOP distribution in the general population is reasonably normally distributed, but not toward the higher pressures, which were the ones that were included in the OHTS simulations. With these limitations, we used as realistic as possible estimates of all involved variables. With these estimates, the incidences of glaucoma in the simulations and the original studies agreed seemingly well (LALES = 4-year incidence simulated 96 of 3681 versus observed 87 of 3772; and OHTS = 5-year incidence simulated 8.6% versus observed 4.4% in the medication group and 9.5% in the observation group). As expected, due to selection based on high measured IOP, the CCT of the participants in the OHTS simulations (mean 599 µm and SD 35 µm; see the Results section) was greater than the CCT of the underlying general population (mean 540 µm and SD 40 µm; see the Methods section). In the original OHTS cohort,^23^ mean (SD) CCT was 573 (39) µm – also clearly greater than that of the general population from which the OHTS participants were recruited, but the difference appears less pronounced than in the simulations. This could be related to the simplifications we made in the simulations (listed above). Both LALES and OHTS adjusted for several possible confounders, including age, in their IOPm-adjusted analysis. In our simulations, we adjusted only for IOPm, to present the effect of collider bias as clearly as possible. Age is formally not a confounding factor in the simulations; age is admittedly strongly associated with the outcome (incident glaucoma) but – in the simulated data – uncorrelated with the predictor of interest, CCT. To explore the effect of also considering age in the analyses, we added age to our IOPm-adjusted models. The results as presented in the right panel of Figure 3 were very similar, whereas the point estimates in the right panel of Figure 4 slightly increased. This slight increase might be due to an increase in precision of the model fit, related to the strong association between age and glaucoma risk (i.e. adjusting for age reduces noise for examining other associations).

Our findings question whether CCT is biologically associated with glaucoma and suggest that current evidence may be explained by collider bias. However, a lack of a biological relationship does not suggest that CCT is not important clinically. In fact, our simulations show that CCT is a powerful way of helping uncover variation in true IOP given measured IOP. So, whereas we would not wish to use CCT alone as a factor to identify people at high risk of glaucoma in the general population, using CCT in combination with measured IOP may be superior to using measured IOP alone. Additionally, in a setting where patients are selected for higher IOP (i.e. in a typical glaucoma clinic), then CCT will also be a helpful clinical parameter, by helping to stratify patients and identify patients whose true IOP may not be raised but just have high measured IOP due to a thicker CCT.

Researchers interested in examining causal relationships need to be aware of collider bias to avoid subsequent spurious interpretation of biased results. Here, we provide some practical steps to avoid such pitfalls. First, it is good practice to identify relevant covariables for the relationship of interest, and determine whether they will be potential confounders or troublesome colliders. A common pitfall is to assume that all covariables associated with the main exposure or outcome are confounding factors which should be adjusted for in multivariable models. However, only by considering the directions of the causal effects rather than simply any associations can researchers reliably differentiate between confounding factors and colliders. As such, drawing a directed acyclic graph at the outset of a study can help identify confounding factors (which should be adjusted for) and colliders (which should not be adjusted for or selected on when examining the causal nature of a relationship). It is also important to decide whether the study cohort is already selected on the basis of a collider. As illustrated in the OHTS example and our simulations, simply selecting on a collider (measured IOP >24 mm Hg in our example) can induce an artifactual crude association between CCT and glaucoma. It is not just adjusting for a collider that induces bias. In this situation, given there is no easy remedy if all study participants are already selected on the collider, it is important to be aware that any significant association may not be causal and state this clearly in the discussion to avoid misinterpretation by others. If the study population is not selected on a collider, but a collider covariable exists in the analytical dataset, researchers must be careful in the construction of multivariable models and the subsequent interpretation of results. A general recommendation is that crude associations are presented in addition to multivariable models. If an association only becomes apparent after adjusting for covariables, then it must be considered whether one or more of the adjusting covariables are colliders and that the observed association is spurious and not causal. It should be noted that collider bias is apparent whether considering variables as continuous traits or as categorized variables (e.g. dichotomized or tertiles). We additionally analyzed our simulations considering CCT as a tertile variable rather than a continuous trait, and the same collider bias was demonstrated (data not shown). Last, it should be stressed that there are situations in which it is appropriate to include collider variables in multivariable models. If the aim of the study is not to examine the biological causality of a relationship, but simply to build the most predictive model of an outcome, then including colliders may be appropriate. For example, in the OHTS scenario, even though CCT is likely not biologically related to glaucoma risk, in a population selected on measured IOP it is significantly predictive of glaucoma (due to the collider bias we have described). Therefore, CCT should be included in glaucoma prediction models as long as they are applied to a similarly selected population (i.e. patients with ocular hypertension), and that causal inferences are not made.
